# Organizational transformation for greater sustainability impact: recent changes in a scientific research infrastructure in Europe

**DOI:** 10.1007/s10980-023-01624-y

**Published:** 2023-04-07

**Authors:** Jennifer M. Holzer, Daniel E. Orenstein

**Affiliations:** 1 Environmental Sustainability Research Centre, Brock University, St. Catharines, ON L2S 3A1 Canada; 2 Faculty of Architecture and Town Planning, Technion - Israel Institute of Technology, 32000 Haifa, Israel

**Keywords:** Long-term socio-ecological research, Social-ecological systems, Organizational change, Sustainability transformations, Knowledge integration, Interdisciplinary, Transdisciplinary, Stakeholder engagement

## Abstract

**Context:**

Scholars across holistic, transdisciplinary, place-based fields of research, such as landscape ecology and social ecology, have increasingly called for an ‘all-hands-on-deck’ approach for transformations toward greater sustainability of social-ecological systems. This Perspective showcases organizational transformation toward sustainability in the context of a research network dedicated to place-based, social-ecological research in Europe.

**Objectives:**

Using the European LTER research infrastructure (eLTER RI) as a case, we analyze recent organizational-level shifts motivated by desires to increase sustainability impact. These shifts include knowledge integration between the natural and social sciences, stakeholder engagement, and a reformulation of administrative guidelines and practices.

**Methods:**

Following a program evaluation, new conversations led to new initiatives in the eLTER RI. As researchers who were involved in the program evaluation and the development of new initiatives, we rely on our professional experience and participant observation to provide insights about this process and its developments.

**Results:**

Recommendations from a recent assessment that critiqued and provided recommendations for the research infrastructure have recently been implemented in the eLTER RI. eLTER has leveraged a unique and timely opportunity—formal recognition and project funding by the EU—to upscale and standardize its infrastructure by creating novel protocols and enacting steps towards implementation.

**Conclusions:**

This Perspective demonstrates how eLTER’s research agenda and related protocols have evolved to better integrate multiple knowledge types, promote stakeholder integration into research, and foster greater equity and reflexivity in doing science, all of which are considered necessary to increase sustainability impact. We conclude by considering current and potential future challenges.

## Calls for more effective transdisciplinary science and the importance of sharing success stories

While scientists, practitioners, stakeholders, and rightsholders (Wilson 2003; Pidgeon 2019) may feel daunted at the range and complexity of threats to sustainability, particularly at the landscape scale (Müller et al. 2010; Opdam et al. 2013), there are scientifically justified reasons for hope emanating from the social-ecological systems (SES) research community. For example, scholars have recently collected a host of examples of bottom-up science-society collaborative initiatives whose activities have led communities towards more positive, sustainable futures (Bennett et al. 2016). This Perspective article uses similar logic and projects similar optimism, recognizing the benefits of learning from “bright spots” (sensu Bennett et al. 2016) at a time when we are coming to terms with the fact that humanity is dangerously exceeding planetary boundaries (Rockstrom et al. 2009; Murphy et al. 2021). In this essay, we offer an example of organizational transformation toward greater sustainability, building on three decades of social-ecological research and innovation in science administration.

Social Ecology, the intellectual tradition with which we identify, aims “to generate the knowledge necessary to understand this [sustainability] crisis and to react to it in the sense of helping establish the ‘ought’ state of societal nature relations” (Fischer-Kowalski & Weisz 2016, p.18). Like Landscape Ecology, Social Ecology studies human–environment interactions at the landscape scale (Hausknost et al. 2016; Dirnböck et al. 2013; Haberl et al. 2006). We view these fields of study as overlapping and complementary; they both adopt holistic, transdisciplinary research approaches and are both concerned with addressing questions of sustainability (Linehan and Gross 1998; Naveh 2000, 2005; Wu and Hobbs 2002; Tress et al. 2004; Wu 2008). The subject matter, scale of analysis, research methodologies and—with particular pertinence for this essay—the normative values and assumptions, are often shared between the two fields, despite evolving via different intellectual histories.

Recent scholarship has enjoined researchers to apply their skills towards addressing the environmental degradation and social inequities that accompany exceeding planetary boundaries (Meadows 1999; Abson et al. 2017). These researchers call for systemic transformations, based on the idea that, in order to catalyze effective change, scientists should focus on addressing deeper societal trends driving environmental degradation (i.e., deep leverage points) rather than trying to change easier, though ultimately superficial, characteristics (Meadows 1999; Abson et al. 2017). Meadows (1999) suggested that acting on such strategic leverage points could have a profound ripple effect on society, leading to broader systemic changes. Along these lines, Abson and colleagues proposed three realms of leverage for sustainability research: strengthening human-nature interactions, reconfiguring organizational dynamics, and sustainability-related knowledge creation and use. Based on these premises, they call for research and knowledge production on “interventions that simultaneously address organizational reform, human-nature interactions, and knowledge productions” (Abson et al. 2017: 9).

Towards the same goal of catalyzing sustainability transformations, scholars have called for greater transdisciplinarity (TD) in research, in which scholars, practitioners, and stakeholders co-produce knowledge about systems in order to collaboratively advance integrated sustainability knowledge of the system and the direct uptake of this knowledge into planning, policy, and management (Schapke et al. 2018; Schneider et al. 2019). SES research is inter- and transdisciplinary by nature and the need for effective collaboration between SES research and practice and stronger science-policy interfaces have been emphasized (Biggs et al. 2021). Results of a study of 31 projects found that practitioners of TD approaches conceptualized the impact of their work in three ways: (1) advancing the knowledge necessary for “…more informed and equitable decision-making, (2) fostering social learning for collective action, and (3) enhancing competencies for reflective leadership” (Schneider et al. 2019, p.26). While these are clearly desirable steps towards sustainability transitions, assessments of long-term systemic change were more ambivalent; TD projects were not able to claim more meaningful impact; this was due to the complexity of the SES in which they worked, the broad diversity of actors and interests affecting the system, and the difficulty of assessing these interacting and overlapping elements.

TD research is only as robust as its ability to incorporate different types of knowledge (e.g., interdisciplinary, practical know-how, Indigenous ways of knowing, etc.) in a single project or endeavor (Godeman 2008; Pohl et al. 2011; Lam et al. 2021; Straub et al. 2021). The integration of natural sciences and social sciences knowledge is a common challenge of transdisciplinary work. One challenge of integration lies in agreeing upon conceptual frameworks and methods that can be used for TD work by diverse collaborators (i.e., can researchers of diverse disciplinary backgrounds speak a common language?). Another challenge lies in the debate between the importance of context-dependent science (Lam et al. 2021) and the need to improve capacity to make generalizations from case study research (Bennett et al. 2021).

In this Perspective, we use the eLTER RI^1^ as a case study to analyze how an infrastructure for SES research can effectively support knowledge integration, more effective stakeholder engagement, and a more equitable, context-aware way of doing science. In particular, we describe how the eLTER RI has initiated a process of actualizing its potential for societal impact, not only by rethinking and recalibrating *what* science is done, but in *how* it is done. We tell this story—of how a large, transnational ecosystem research network began to internalize emerging knowledge regarding the benefits of inter- and transdisciplinarity, and how it has begun to institutionalize particular TD values into its administrative structure and research program.

We will elaborate on how eLTER has incorporated a new conceptual framework, ethical commitments, assessment protocols, stakeholder engagement approaches, and other measures intended to support TD research toward sustainability transformations. These changes can be seen to constitute “interventions that simultaneously address organizational reform, human-nature interactions, and knowledge productions” (Abson et al. 2017: 9), and therefore offer important lessons for those interested in organizational transformations toward sustainability. We show how program changes constitute the seeds of organizational transformation (Baker-Shelley et al. 2017). In doing so, we highlight the necessity of holistic shifts in thought and action, from changes in organizational policies to changes in the way individuals think, communicate, and conduct research. We hope that sharing this story will provide examples and inspiration for others seeking to change the status quo in social-ecological science at the landscape scale.

We, the authors, have both played roles in the eLTER RI by carrying out an EU-funded audit of LTSER research platforms (2016–2020; Holzer et al. 2018a, b; Holzer et al. 2019). DEO has held a leadership role in preparing the PPP and PLUS grants discussed below, as well as advancing some of the strategic documents outlined in the third section below. We believe that our intimate engagement with the RI and the processes described in this paper enable us to share details that improve the transparency of what is often an “insider” process. In the spirit of TD research, we attempt to be self-aware, introspective, and critical when necessary, in order to provide a candid and effective critique of the described organizational transition.

## The roots of LTER and early efforts to integrate social ecology

The US LTER network was founded in 1980; its primary focus during its initial years was on ecosystem properties and their biophysical variables (Aronova et al. 2010). If human aspects were integrated into LTER research, it was primarily via studies of the negative impacts of human activities on ecosystem function. In the 1990s, US LTER scientists discussed integrating the social sciences into the network’s research program, but tangible activities were not immediately initiated (Redman et al. 2004). In its 2002 review of LTER, the US National Science Foundation (NSF) made 27 recommendations, including an explicit call for LTER to collaborate with social scientists, the establishment of cross-site projects, and a focus on synthesis science (NSF 2002; Redman et al. 2004; Mirtl 2010). Redman and colleagues (2004) therefore suggested a conceptual framework for integrating activities and delineated key interdisciplinary questions for LTER to consider. Their approach advocated addressing questions of societal importance which were associated with long-term ecosystem processes, and which were better studied across a network of sites. Over the next two decades, social dimensions were integrated into two urban LTER sites in the US and elsewhere, ultimately contributing to the development of novel frameworks for transdisciplinary research and the study of urban socio-ecology (Grove & Pickett 2019, 2021).

The NSF, as a key mentor of LTER activities, was intent on making the global LTER network independent of US LTER (Mirtl 2010). An international LTER (ILTER) network was established in 1993. Its focus was initially on long-term ecosystem observation, but it grew to engage in “site-based ecological and socioeconomic research,” representing a powerful network of ecosystem research facilities comprising 40 national member networks by 2008 (Mirtl 2010).

In 2001, concurrent to the US LTER review, a European Environment Agency (EEA) report critiqued the fragmentation of ecosystem research in Europe and called for stronger links between ecosystem research and monitoring (i.e., Gee 2001, as cited in Mirtl 2010). LTER—Europe, or “eLTER” as it is called today, evolved out of a European-funded Network of Excellence, ALTER-Net. Although eLTER’s conceptual research framework embraced an SES approach from its inception, implementing this approach has proven challenging.

Within eLTER, SES research was organized around Long-Term Socio-Ecological Research (LTSER) “platforms”. These platforms are geographical areas that typically encompass classic LTER sites, but also include broader geographic regions, thereby integrating key cultural, administrative, historic, economic and other social dimensions. An SES approach was further advanced by a series of publications which advocated for a comprehensive shift from LTER to LTSER, set out the theoretical justification for this shift, developed a blueprint for the physical structure of LTSER platforms, and presented case studies of LTSER platforms (Haberl et al. 2006; Singh et al. 2013). While on-the-ground research and ecosystem observation continued to focus primarily on natural sciences expertise and methodological approaches over the next decade, numerous training workshops and exploratory research projects were conducted to strengthen TD competencies of eLTER scientists.

During the years 2014–2018, with generous funding of a European H2020 grant, eLTER conducted a comprehensive audit of its capacities to conduct SES research and its output. Results, summarized in a trio of articles by the current authors (Holzer et al. 2018a, 2018b, 2019) and others (Gingrich et al. 2016; Dick et al. 2018; Angelstam et al. 2019) yielded the following observations and recommendations, among others:

1. Observation: LTSER platform leaders strive to do transdisciplinary SES research, but platform infrastructure lacks frameworks, capacities, and training to enable effective TD work.

*Recommendation:* LTSER platforms can begin to address this issue by better leveraging the benefits of network membership, such as harmonized datasets, site access, long-term funding, and planning for a coordinated research agenda with local needs for data, knowledge, and relationship-building with stakeholders.

2. Observation: Platforms were reported to be dominated by ecosystem research (72%), with only 28% social research, and platform research programs were typically maintained by 3–5 staff members (Angelstam et al. 2019).

*Recommendation*: Strengthen the role of the social sciences and humanities, encourage macroecological approaches, and strengthen stakeholder participation. Increase knowledge exchange, reciprocity, and responsiveness between (a) interdisciplinary scientists, particularly social scientists and natural scientists, and (b) scientists and other stakeholders and interfacing with other landscape approach concepts.

LTSER platforms should start organizational change processes by clearly defining objectives (e.g., through writing and publishing memoranda of understanding), outlining protocols (e.g., for defining the research agenda, for how to engage non-academic stakeholders, etc.), and clearly defining roles for personnel.

These findings and recommendations (Table 1) highlight the LTSER platform as a key infrastructure for conducting SES research in Europe. Further, the findings imply that an SES approach does not just advocate adopting a novel research field or agenda, but also requires an alignment of underlying values (e.g., towards inclusivity and integration of new, and sometimes non-scientific, knowledge sources), commitments towards working with stakeholders and building new partnerships, and an adjustment of research agendas to fit stakeholder needs at the landscape scale. In subsequent iterations of eLTER development, the importance of these aspects for advancing LTSER platforms in Europe were recognized by eLTER coordinators and served as triggers for change; we discuss these transformations below.

**Table 1:** Assessment findings and recommendations. Adapted from Holzer et al. 2019

Area of inquiry	Findings	Recommendations
What added value do LTSER platforms offer to society?	Problem-solving orientation Connection to global research network New collaborations, broader participation Promotion of long-term research and observation	1. Clearly articulate and publicize the mission, objectives, and expectations for research, monitoring, and other activities at the platform scale, including defining the end-users of knowledge products and time frame for decisions. Publicizing activities and achievements may help build partnerships. 2. Define roles for researchers and partners. Hire staff dedicated to coordinating platform activities. Reward stakeholders for contributing their time. 3. Align goals for long-term research and observation; this may help buffer temporary shortfalls in resources to sustain long-term ambitions. 4. Improve knowledge exchange and accessibility of data and research findings, including database maintenance. Knowledge exchange activities should fit local culture and participation should be incentivized. 5. Incorporate social scientists, community leaders and administrators into platform management. Aim for diversity in LTSER leadership, including citizen science and school programs. 6. Understand and define target scales for projects to make wise use of collaborators’ time. 7. Initiate structured, periodic evaluation to foster continuous learning and improvement. Incorporate reflection, self-assessment, and participatory approaches.
How well do LTSER platforms integrate the social sciences with natural sciences research?	Demand for more social science research Existing research integrated in a piecemeal fashion Power asymmetry between natural scientists and social scientists	
How significantly are stakeholders involved in research processes?	Research primarily initiated by scientists Some educational and citizen science initiatives	
What are the impacts of research associated with LTSER platforms?	Research outputs (e.g., academic articles and reports) Knowledge exchange At each LTSER platform, charismatic scientists act as champions, exploiting opportunities to further LTSER platform mission and goals	
What are the key challenges faced by LTSER platforms?	Working collaboratively with stakeholders / end-users to define research objectives and conduct relevant, actionable science Dedicating resources to transdisciplinary knowledge integration, mobilization, and translation Leadership and guidance to tackle inertia and change ways of conducting science	

## The path to sustainability: eLTER doubles down on transdisciplinarity

### Formalizing strategic features of eLTER RI’s transdisciplinary research program

Strategic features are elements of a long-term research platform that are “designed and deployed to support multi-sectoral, interdisciplinary, and transdisciplinary collaborations” (Grove & Pickett 2019). They depend upon multiple sectors and disciplines, and are used to create communities, data, and knowledge systems (Grove & Pickett 2019). This section narrates how eLTER has used recent opportunities to formalize strategic features that have been in formation for some time to advance desired outcomes. Throughout its history, ILTER and eLTER have emphasized that their greatest potential contribution to global sustainability is through their research and data (Mirtl et al. 2018 Fig. 1). But these networks also internalized as axiomatic that a sustainability agenda could be best served through the adoption and implementation of a particular way of doing research, i.e., social-ecological, transdisciplinary research. Yet, the audit of eLTER’s SES agenda described above revealed a significant gap between its aspirations and its implementation.

**Fig. 1:**
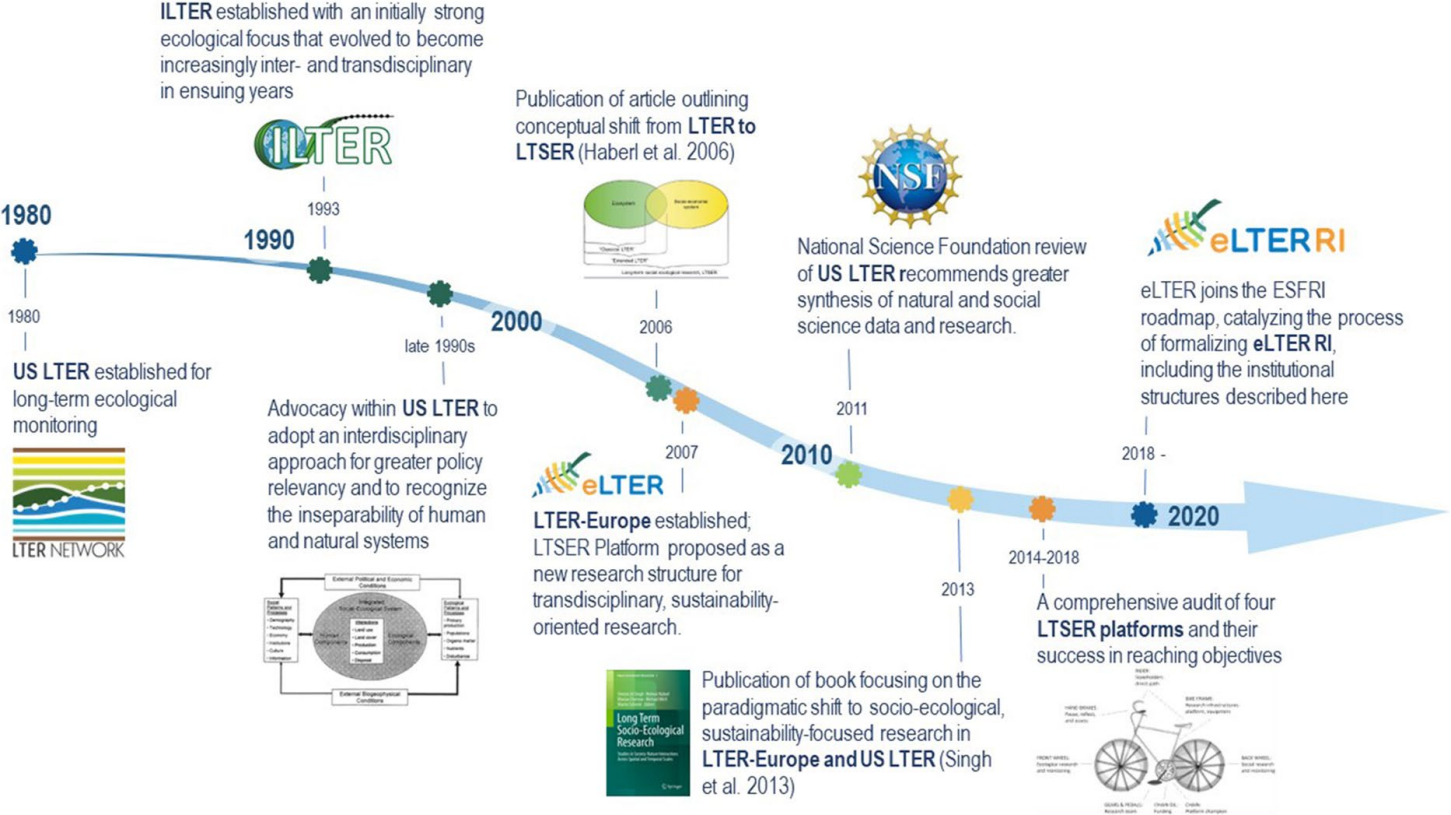
Major milestones in the evolution of eLTER RI’s organizational orientation strengthening its potential for sustainability impact. Information compiled from Redman et al. 2004, Mirtl and Krauze 2007, Aronova et al. 2010, Knapp et al. 2012, Vanderbilt and Gaiser 2017, Mirtl 2018a, 2018b, Mirtl et al. 2018, and Dick et al. 2018. Design: Ronit Cohen-Seffer

In 2018, eLTER took the opportunity of being accepted into the European Strategy Forum on Research Infrastructures (ESFRI) to institute broad and deep changes in its physical, administrative, and scientific structure. ESFRI is an advisory body to the European Union (EU) which seeks to strengthen the science-policy interface through the development of scientific research infrastructures.^2^ By being accepted into the ESFRI “Roadmap”, eLTER has received institutional support to significantly strengthen its facilities and research and observational capacities. Concurrent to joining the ESFRI Roadmap, eLTER revised its full title to emphasize its whole systems approach: “eLTER—Integrated European Long-Term Ecosystem, Critical Zone and Socio-ecological System Research Infrastructure”. The description defines the mission of the eLTER Research Infrastructure (RI, its formal title as part of the ESFRI Roadmap), as “integrating traditional natural sciences and holistic ecosystem research approaches, including studies of the human component, to better understand ecosystems”.^3^

Acceptance to the ESFRI Roadmap favorably positioned eLTER RI to win H2020 grants for two large-scale projects (starting in 2019)—eLTER Preparatory Phase Project (PPP) and eLTER Advanced Community Project (PLUS). Though interacting, these two projects differ substantively in their content, with PPP focusing on establishing the legal, financial, and technical specifications of the RI, and PLUS enabling proof-of-concept research based on the conceptual approach and physical capacities of the RI. To date, proposal-writing and subsequent project implementation has afforded eLTER the opportunity to make another strong push towards fuller integration of TD principles into RI activities, and realization of the underlying values embodied in transdisciplinarity. eLTER has made formal commitments to leveraging science in the service of sustainability through its PPP and PLUS grants, thereby highlighting its potential to contribute to Europe meeting its Sustainable Development Goals (SDGs). The RI’s SES research is touted as the primary mechanism to realizing its contribution to sustainability, but, as we demonstrate below, eLTER is building an array of tools and mechanisms into all the details and processes of doing science, through which, it is hoped, it can maximize its contribution to sustainability.

### A new vision made real through new documents: strategic plan, ethical frameworks, and formative assessments

**Fig. 2:**
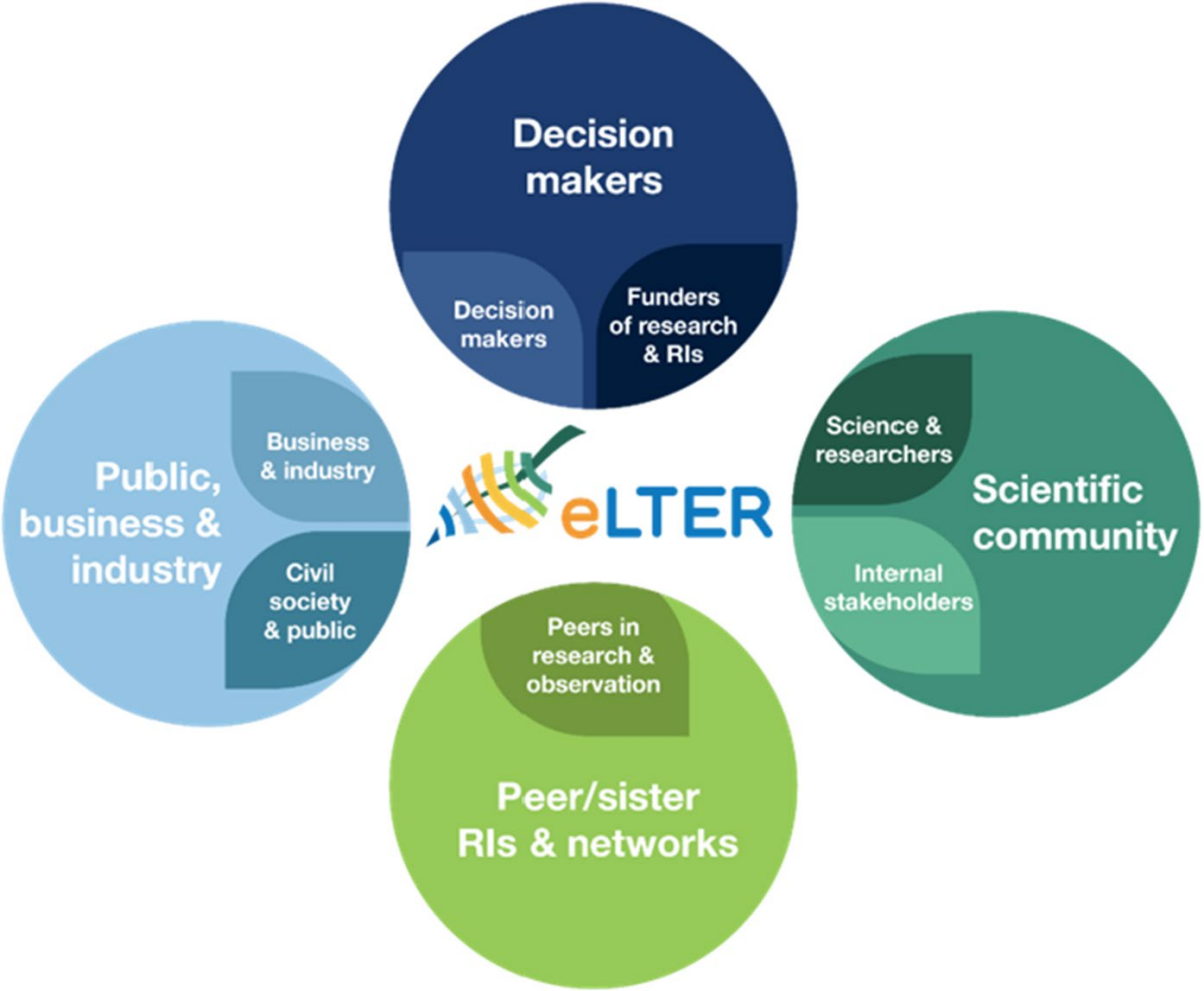
Stakeholder mapping of eLTER RI (from Barov et al. 2021)

What follows is an inventory of conceptual frameworks, tools, and mechanisms recently developed as part of the institutionalization of eLTER RI to focus and support the RI’s efforts to pursue its objective to help foster societal sustainability transitions. Most of these are noted explicitly in eLTER’s newly adopted strategic plan (Nikolaidis et al. 2021), and each is currently being further developed and documented as PPP and PLUS deliverables. It is important to note that these tools and mechanisms for change were developed in a highly iterative process of collective discussion, consultation, debate, and compromise across multiple stakeholder groups (Fig. 2).

#### Grand challenges

In its proposals, strategic plan, and major publications, eLTER designates four global ecological challenges, derived from the EUs 7th Environment Action Programme and other global calls, to frame its research endeavors. They include (1) biodiversity loss; (2) climate change adaptation and mitigation; (3) food security and threats to soil and water; and (4) sustainable management of natural resources. Accordingly, in eLTER PLUS, each one of these themes was assigned an RI scientist to serve as a “theme lead”, to assure that RI research, data collection, and community engagement focus on these four themes and integration between them, and to promote knowledge products to the policy community and the public.

#### Whole systems approach

In its far-reaching effort to demonstrate its commitment to interdisciplinarity, eLTER conceptualizes social and biophysical systems into a single SES with multi-directional feedbacks, together with Critical Zone research, which links the disciplines associated with water, air, life, rock, and soil research (Waldron 2020). Accordingly, each of its research, monitoring, and data services is developed within this holistic perspective (Mirtl et al. 2021).

#### Strategic plan

A strategic plan succinctly clarifies the organization’s *raison d’etat*, its goals and objectives, and the paths through which the organization intends to reach those goals. As the organization evolves, a strategic plan serves as a reference point for where the organization would like to go and what it intends to achieve. eLTER’s strategic plan (see https://elter-ri.eu/), adopted in 2021, sets out its institutional vision to use ecosystem science and research in service of environmental sustainability. “Environmental sustainability can only be achieved on the basis of the robust knowledge and empirical evidence needed to identify and mitigate human impacts on ecosystems. eLTER catalyzes scientific discovery and insights through its state-of-the-art research infrastructure, collaborative working culture, and TD expertise. This enables the development and application of evidence-based solutions for the wellbeing of current and future generations” (Nikolaidas et al. 2021). In other words, eLTER is committed to developing a research infrastructure that will contribute to sustainability not only through data and research products, but also via institutional operating procedures (i.e., its values, ethics, and behaviors). All of the elements included in this list are introduced in the strategic plan as organic to the infrastructure’s identity and working culture.

#### Ethical framework

Promoting inclusive societies free of discrimination and reducing inequalities are ubiquitous themes across all of the UN’s SDGs (e.g., 13 of the 17 goals refer to social equity and inclusiveness; Gupta & Vegelin 2016). Recognizing the tight link between environmental sustainability, on the one hand, and values and ethical conduct, on the other, eLTER has invested in defining a wide-reaching and ambitious ethical framework. In April 2021, eLTER RI published its Gender Equality Program, which included clear commitments to address systemic and pervasive prejudices faced by women in the scientific and academic communities, and outlined a series of actions that eLTER will take to address these issues within the infrastructure and beyond. In particular, it pledges parity in gender representation in decision-making positions and scientific leadership, and implements internal mechanisms to address gender bias in eLTER and to educate towards inclusivity (Orenstein et al. 2021).

In October 2022, eLTER RI published its Ethical Guidelines (Orenstein et al. 2022) which expanded eLTER’s commitment to work for greater inclusion and prevent discrimination to include all demographics (e.g., race, nationality, religion, gender identity, and more), as specified in the European Charter of Fundamental Rights (European Union 2012). In order to assure these commitments are fulfilled, a volunteer gender equity and non-discrimination ombudsperson position was created to provide an address for potential complaints, and to oversee education programming for eLTER staff and researchers. The Ethical Guidelines also include sections on (1) research process and conduct, (2) data collection, management and dissemination, and (3) organizational environmental performance, each with performance criteria to assure compliance. Finally, the Ethical Guidelines provide for the creation of an Ethical Advisory Board to assess progress and provide oversight.

Since 2020, eLTER has given explicit public expression to its ethical commitments, including official statements regarding eLTER’s potential contribution to assessing COVID-19 impact^4^ and its condemnation of the 2022 violent invasion of Ukraine.^5^ eLTER has also issued public announcements and organized events commemorating International Day of Women and Girls in Science, and International Women’s Day.^6^

#### The [holistic] socio-economic impact assessment

TD theory emphasizes self-reflection, both as a means to consider the effectiveness of teamwork and collaboration and the impacts of research activities on outcomes, and also as a mechanism for empowering stakeholders in the process of knowledge co-production (Haberl et al. 2006). Regarding research impacts, eLTER RI will implement a periodic review process in which the impacts of its activities are regularly assessed according to predefined indicators selected to reflect three levels of impact (outputs, outcomes, and long-term impact) for six categories defined in the strategic plan (data services and flow, scientific excellence, stakeholder engagement, cooperation with civil society and private sector actors, training, and conducting societally-relevant SES research). This reflects eLTER’s commitment to hold itself both to high standards for scientific, social, and economic impacts, and also to address ESFRI’s expectations for RIs accepted onto the Roadmap. Within this assessment framework, indicators will assess transdisciplinarity (e.g., working with stakeholders and greater interdisciplinary engagement), giving RI researchers, administrators, and other stakeholders the tools to determine whether, beyond the documents and good intent, a sustainability transition is actually happening in the RI.

#### Integrated governance and stakeholder engagement

TD work in general, and SES research in particular, is predicated on the assumption that research and its outputs can make a stronger contribution to sustainability when conducted in collaboration with stakeholders (Haberl et al. 2006; Holzer et al. 2019; Biggs et al. 2021). Within eLTER’s PPP project, comprehensive stakeholder mapping was conducted in which stakeholder communities were defined at multiple spatial scales (local, national, and European). This was done with the explicit goal of developing participation channels and forums for stakeholders to provide input on eLTER’s research agenda, and to tailor data collection and services to the specific needs of stakeholder communities (Fig. 2; Barov et al. 2021). Further, various advisory committees were formed and consulted on eLTER’s progress and recommendations from these stakeholders were collected, processed, and integrated into decision making processes. These advisory committees include: the Scientific Advisory Board, the Interim Council, and the Site and Platform Managers Forum, each of which contributed input into the formulation of eLTER policies, which were substantially modified according to this input. Stakeholder engagement at the local and regional scale is a fundamental, explicitly recognized component of LTSER platform operation, eLTER RI’s spatial units for SES (Haberl et al. 2006; Orenstein et al. 2019; Grove & Pickett 2019).

#### Institutionalizing socio-ecological platform structure and objectives

While the idea of dedicated landscape-scale platforms (i.e., geographical areas) in which to conduct SES research has been a prominent feature of eLTER for almost two decades (Haberl et al. 2006; Singh et al. 2010; Mirtl et al. 2013), the 2018 assessment of LTSER platforms described above revealed a loosely-coordinated set of platforms across Europe conducting an eclectic mix of activities of varying import and impact (Holzer et al. 2018b, 2019). eLTER PPP and PLUS set out to make order in the RI and strengthen the impact and longevity of the LTSER platform structure by developing strict parameters by which a platform is recognized and how it operates. This includes setting standards for program elements such as: (1) which data sets are mandatory or optional, and how these data are collected, stored, and made accessible to stakeholders (eLTER “Standard SES Observation Variables” and its service portfolio for the provision of SES variables and tools for data analysis; Peterseil et al. 2020); (2) staffing platforms with the necessary skill sets for both SES research and stakeholder engagement, and; (3) the necessity of a memorandum of understanding to specify which organizations are responsible for overseeing platform research and operations and which stakeholder groups are partnered with the platform. Importantly, these criteria—which are currently being established in parallel for long-term ecological research (LTER) sites—treat the SES research within eLTER on par with its ecological observations and research program. If the recent push for institutionalizing the LTSER platform succeeds, then SES research within eLTER will no longer be a ‘side project’, but integral to all of eLTER’s operations.

Metzger and colleagues (2010) assessed the geographic coverage of LTSER platforms in Europe, identifying a lack of geographical representativeness of LTSER platforms, specifically regarding socio-ecological systems and urban and disturbed regions. They also identified a persistent bias in favor of traditional ecological research (reconfirmed by Holzer et al. 2019) and noted that Mediterranean and Iberian landscapes received relatively little attention (Metzger et al. 2010). Nearly a decade following that study, Mollenhauer and colleagues (2018) conducted another study to determine the SES coverage of eLTER RI. While they found that Mediterranean regions continued to be under-represented in the RI, they also concluded that there had been improvement due to “the impact of strategic efforts made by LTER-Europe in recent years to (i) inform and encourage national LTER site network developments to close gaps or (ii) support the development in countries located in underrepresented areas (e.g. eastern Mediterranean area, LTER Greece)” (Mollehnauer et al. 2018. p. 976). Incidentally, in a separate analysis of global distribution of ILTER sites, Wohner and colleagues found over-representation in Mediterranean zones and areas of high economic density (Wohner et al. 2021), a gap identified at the European, though not the global, scale. The RI can and should continue to identify gaps in spatial coverage and encourage filling gaps through establishment of new sites and platforms, or co-locating research sites and platforms with sibling RIs and research initiatives, such as the Programme on Ecosystem Change and Society (PECS),^7^ Natura and its affiliated organizations^8^ and UNESCO Biosphere Reserves.^9^

#### Increasing relevance to diverse stakeholder communities

As of this writing, eLTER RI is only two years into major projects to develop the RI, so measuring the impact of these innovations would be premature. However, innovative tools to strengthen engagement and integration of stakeholders are already in development. For example, eLTER scientists have introduced two online tools that enable interested individuals to access and process (a) socio-economic and demographic statistical data and (b) bio-physical data extracted from satellite sensors specific to LTSER platforms across Europe (called “cookie-cutting” tools), thereby facilitating SES research at the platform scale and at the cross-platform, continental scale. Further efforts are being invested in a continental-scale citizen science initiative to track biodiversity across platforms via the online app *iNaturalist*. These examples illustrate how eLTER is prioritizing engagement in science by stakeholders and the public.

## Discussion: infrastructure changes signal organizational transformation

**Fig. 3:**
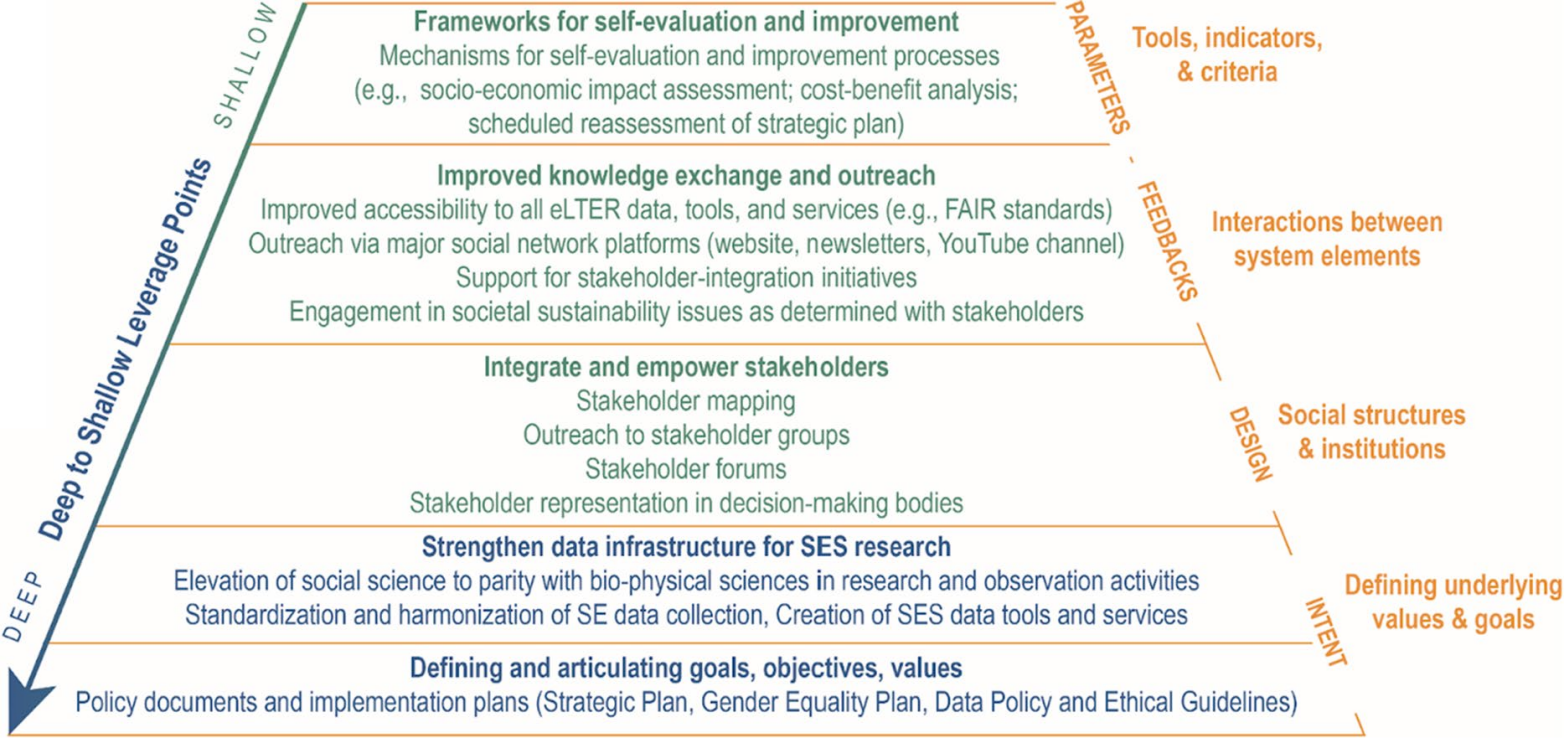
Conceptualization of eLTER’s efforts towards organizational sustainability transformation (following Abson et al.’s (2017) leverage points framework). Design: Ronit Cohen-Seffer

We claim here that the eLTER RI provides a real-time example of an organization undergoing a significant change to strengthen its potential contribution to a broader, societal sustainability transition (Fig. 3). Organizational change happens simultaneously at the individual, organizational and extra-organizational levels (Baker-Shelley et al. 2017). This type of significant change requires an array of actions, from cultivating new skills and competencies of members to implementing new practices across portfolios (e.g. research, professional development, operations, governance, communications) in a holistic manner, to applying relevant external standards to the organization (Baker-Shelley et al. 2017). The changes in eLTER RI policies detailed above encourage participating scientists to adapt their outlook and adjust the process of doing science—to be more inclusive, to include non-scientist stakeholders in meaningful and appropriate ways, to think ahead about the intended impacts of research, to engage is periodic self-assessment regarding gaps between activities and objectives, and to plan for ever-present uncertainty and risk.

A clear, empirically-based foundation for supporting these changes is based on diverse bodies of literature, including SES research, organizational change, and more. However, these “upgrades” are not entirely due to the aspiration to fulfill the recommendations of sustainability research; they also fulfill the practical requirements of EU funders (H2020, EU, ESFRI) that set requirements for stakeholder integration, gender equality, transparency, and other issues (see, e.g., “Ethics—H2020 Online Manual”,^10^ which refers to the EU Charter of Fundamental Rights). However, while eLTER leadership may have initiated these aspects of science because they are mandated, they have stated that they envision that these organizational changes will improve research output and actionability toward addressing grand sustainability challenges (e.g., Mirtl et al. 2018; Orenstein et al. 2019; Nikolaidis et al. 2021). eLTER’s current efforts reflect a process of formalization of its conceptual framework and ethical values. Formalization is important for triggering shifts in how individuals think about TD in their scientific work, for serving as external motivation when old habits creep back in, and for creating boundary objects (e.g., gender equality plan, strategic plan, etc.) that partners and participants can refer to moving forward (Grove & Pickett 2021). Importantly, the commitments described above are “living documents” that will be subjected to periodic review and assessment to facilitate adaptation to changing circumstances. The eLTER RI leadership can attest to the growing pains that come with changing ways of thinking and collaborating; but it is often through a bit of discomfort that individuals realize changes they need to make to become more effective collaborators (Freeth & Caniglia 2020).

### Challenges of realizing these strategic priorities

The aspirational objectives outlined here, along with the tools to actualize them, does not diminish the importance of reflecting upon present and future challenges. First among the challenges is executing effective long-term, transdisciplinary social-ecological research in the field. The challenge of producing transdisciplinary, policy-relevant research and maintaining it for extended time periods was analyzed by Holzer and colleagues (2019), and the challenges described there, presented here in Table 1, continue to be relevant today. eLTER RI scientists continue to search for and initiate “proof-of-concept” research that not only reflects that TD research is actually happening, but exemplifies the success of the approach in contributing to landscape-scale sustainability. In other words, examples of research that (1) engages stakeholders meaningfully, (2) conducts policy-relevant research, and (3) shows desirable socio-ecological impacts are few, but eLTER is taking steps to grow in this area. One productive working example that is exceptional within the RI is research in the French Zone Atelier Plaine et Val de Sévre, which exemplifies all three of these elements (e.g., Bretagnolle et al. 2019; Gaba and Bretagnolle 2020; Berthet et al. 2022). As outlined in Berthet et al. (2022), platform scientists from Zone Atelier Plaine et Val de Sévre have been working closely with farmers for nearly three decades, during which their research program has become increasing holistic, evolving from a purely ecological perspective to one that also focuses on socio-ecosystem dynamics. This research group has expanded its focus on a broadening range of agro-ecological and biological conservation issues, and involved a wider range of scientists and stakeholders. Reproducing this success across eLTER RI would profoundly advance the RI’s sustainability objectives and increase its relevance for European policy-making.

A second challenge is realizing eLTER’s formalized ethical commitments. For example, the RI has been engaged in intense debate regarding the environmental impact of flying, but after cessation of travel for two years during the COVID 19 pandemic, there has been a strong desire to re-establish close working relationships that distanced during the pandemic. Compromises are being tested to reduce the amount of flying; for instance, by holding regional meetings with smaller work teams and setting centrally-located meeting venues with easy access by ground-based transportation. Nonetheless, flying is still considered an absolute necessity by many RI scientists. The first meat-free meeting was also not received positively by many of the workshop participants when the approach was tested, but the RI continues to reduce the amount of meat (and disposable dishes) at its meetings. For most of the other ethical commitments, more time will be needed to see if the guidelines and the tools for their implementation will be successful.

## Conclusion: will greater transdisciplinarity lead to greater sustainability?

We recognize that implementing the ambitious, holistic approach outlined in this Perspective is beyond the capacity of most individual scholars. It necessitates a collaborative network with effective communication. Conducting effective team science is an ever-present challenge for eLTER, as it is for TD science in general. This challenge encompasses: cultivating teams with a common language and accessible boundary objects to communicate effectively, constant vigilance, renewal, review and self-assessment, introspection, frequent reminders that we are meant to do things differently, and guidelines and leadership to integrate knowledge when individuals begin to revert back to their individual expertise. The eLTER experience shows that significant opportunities to advance these efforts arise when they are explicitly required by granting and government agencies.

While only three years have passed since the 2019 assessment findings were shared, eLTER leadership has institutionalized an SES research approach by defining criteria for platform establishment and operation, defining essential SES variables, and developing tools to compile and disseminate data freely and easily. They have begun to document commitments to TD principles, including a strategic plan and eLTER ethical guidelines. These changes magnify the potential of eLTER to contribute to sustainability goals, through its institutional structure and ethical commitments, through the production of knowledge and data, and through partnerships with stakeholders that can magnify this work.

It is too early to tell whether these organizational changes will directly spur sustainability transitions in LTSER platforms, but eLTER as a network of scientists and stakeholders, scientific infrastructure, and knowledge production is already a more resilient institution (see, e.g., definition of institutional resilience in Steinberg 2009) as a result of the due diligence, reflexivity, and tough conversations that are shifting the status quo of doing SES science across Europe.

## Footnotes

1 Integrated European Long-Term Ecosystem, critical zone and socio-ecological Research (https://elter-ri.eu/).

2 https://www.esfri.eu/about

3 http://roadmap2018.esfri.eu/projects-and-landmarks/browse-the-catalogue/elter/

4 https://elter-ri.eu/news/role-environmental-ris-context-current-covid-19-crisis-position-elter

5 https://elter-ri.eu/statement-violence-ukraine

6 https://elter-ri.eu/news/elter-celebrates-international-womens-day-2022

7
https://pecs-science.org/.

8 https://natura-net.org/.

9 https://en.unesco.org/biosphere.

10
https://ec.europa.eu/research/participants/docs/h2020-funding-guide/cross-cutting-issues/ethics_en.htm.
